# Histogram analysis of mono-exponential, bi-exponential and stretched-exponential diffusion-weighted MR imaging in predicting consistency of meningiomas

**DOI:** 10.1186/s40644-023-00633-z

**Published:** 2023-12-05

**Authors:** Lingmin Zheng, Peirong Jiang, Danjie Lin, Xiaodan Chen, Tianjin Zhong, Rufei Zhang, Jing Chen, Yang Song, Yunjing Xue, Lin Lin

**Affiliations:** 1 Department of Radiology, Fujian Medical University Union Hospital, Fuzhou, 350001 China; 2 Department of Radiology, Clinical Oncology School of Fujian Medical University, Fujian Cancer Hospital, Fuzhou, 350014 China; 3 Department of Neurosurgery, Fujian Medical University Union Hospital, Fuzhou, 350001 China; 4 MR Scientific Marketing, Healthineers Ltd, Siemens, Shanghai, China; 5 School of Medical Technology and Engineering, Fujian Medical University, Fuzhou, 350004 China

**Keywords:** Tumour consistency, Meningioma, Diffusion MRI, Bi-exponential model, Stretched-exponential model

## Abstract

**Background:**

The consistency of meningiomas is critical to determine surgical planning and has a significant impact on surgical outcomes. Our aim was to compare mono-exponential, bi-exponential and stretched exponential MR diffusion-weighted imaging in predicting the consistency of meningiomas before surgery.

**Methods:**

Forty-seven consecutive patients with pathologically confirmed meningiomas were prospectively enrolled in this study. Two senior neurosurgeons independently evaluated tumour consistency and classified them into soft and hard groups. A volume of interest was placed on the preoperative MR diffusion images to outline the whole tumour area. Histogram parameters (mean, median, 10th percentile, 90th percentile, kurtosis, skewness) were extracted from 6 different diffusion maps including ADC (DWI), D*, D, *f* (IVIM), alpha and DDC (SEM). Comparisons between two groups were made using Student’s t-Test or Mann-Whitney U test. Parameters with significant differences between the two groups were included for Receiver operating characteristic analysis. The DeLong test was used to compare AUCs.

**Results:**

DDC, D* and ADC 10th percentile were significantly lower in hard tumours than in soft tumours (P ≤ 0.05). The alpha 90th percentile was significantly higher in hard tumours than in soft tumours (P < 0.02). For all histogram parameters, the alpha 90th percentile yielded the highest AUC of 0.88, with an accuracy of 85.10%. The D* 10th percentile had a relatively higher AUC value, followed by the DDC and ADC 10th percentile. The alpha 90th percentile had a significantly greater AUC value than the ADC 10th percentile (P ≤ 0.05). The D* 10th percentile had a significantly greater AUC value than the ADC 10th percentile and DDC 10th percentile (P ≤ 0.03).

**Conclusion:**

Histogram parameters of Alpha and D* may serve as better imaging biomarkers to aid in predicting the consistency of meningioma.

**Supplementary Information:**

The online version contains supplementary material available at 10.1186/s40644-023-00633-z.

## Background

Meningioma is one of the most common primary intracranial tumours [1]. With surgical resection as the treatment of choice for meningiomas, the consistency of meningiomas is crucial for surgical planning and prognosis. Soft meningiomas can be easily removed by only suction with minimal invasion to the surrounding normal tissue. However, harder tumours are usually more difficult to remove and require additional instruments (ultrasonic aspiration and scissors), prolonging the operation time and increasing blood loss. This may lead to a higher rate of subtotal resection and perioperative complications [2]. Therefore, if hard consistency can be accurately predicted before surgery, a more experienced surgical team, better prepared blood and more extensive neurophysiological monitoring can be prepared to optimize surgical strategies and obtain a more favourable prognosis.

At present, several histological factors have been identified to be related to hard tumour consistency, such as high cellularity [3], rich fibrous components [4, 5], and low vascularity [5–7]. Nevertheless, this valuable histological information can only be obtained after surgery or biopsy. Alternatively, diffusion magnetic resonance imaging (MRI) is a non-invasive method to assess tumour biology and quantify physiological processes [8, 9]. The apparent diffusion coefficient (ADC) value obtained from mono-exponential model (MEM)-based diffusion-weighted imaging (DWI) is the most frequently used and studied diffusion metric to evaluate tumour consistency [10–14]. However, the results of these studies vary. ADC was found to be either lower [10, 11] or higher [12] in hard meningiomas than in soft meningiomas. Other studies even found no association between ADC and meningioma consistency [13, 14]. The conflicting results may be attributed to the fact that the ADC only reflects the overall water diffuse motion, such as water molecular diffusion and blood microcirculation [15] and fails to accurately represent specific tissue characteristics. Bi-exponential model (BEM)-based DWI reflects the separation of simple diffusion and microvascular perfusion by yielding 2 perfusion-related metrics: pseudo-diffusion coefficient (D*) and perfusion fraction (*f*) [16]. D* macroscopically describes the incoherent movement of blood in the microvasculature compartment, and *f* denotes the fraction of the incoherent signal that arises from the vascular compartment in each voxel over the total incoherent signal. However, tissue characteristics cannot be fully explored by dividing water diffuse motion into only two compartments [17]. Stretched exponential model (SEM)-based DWI is an advanced diffusion imaging method that considers the composite of continuous distribution of ADCs in each part. Therefore, SEM can reflect tissue characteristics by calculating the heterogeneity of intravoxel diffusion rates and the distributed diffusion effect within each voxel in multiple pools of water molecules [18]. BEM and SEM have been widely explored in studies of meningiomas [19–22] to assess histological grade, subtype, vascular density and differentiability [22–24]. However, their application in predicting meningioma consistency is still lacking. Meanwhile, intratumoural heterogeneity is another possible reason for the inconsistent results of meningioma consistency prediction. Voxel-based whole tumour histogram analysis is an objective and reproducible method that provides a myriad of information and details inside the tumour based on the tissue volume [25]. Hence, the histogram parameter is expected to reflect the overall heterogeneity of the tumour.

As advanced diffusion MR models, BEM and SEM can provide more information relative to tumour histological components and structures, while histogram parameters can reflect the intratumoural heterogeneity. We hypothesized that advanced diffusion MR models with histogram analysis can better predict meningioma consistency than the traditional diffusion model (MEM). Hence, the current study aimed to evaluate and compare the potential of various histogram parameters extracted from different diffusion maps obtained from the MEM, BEM, and SEM-based DWI in predicting the consistency of meningiomas.

## Methods

### Patients

**Fig. 1:**
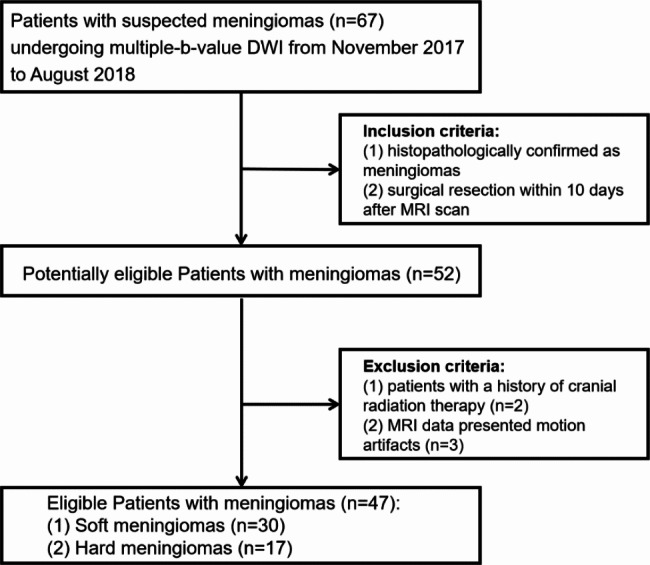
Patient enrolment flowchart

The present study was approved by the local institutional review boards. Written informed consent was obtained from all patients before participation. Sixty-seven consecutive patients with suspected meningiomas in our hospital from November 2017 to August 2018 were prospectively included according to the following inclusion criteria: (I) performed preoperative brain MRI including multiple-b-value DWI; (II) received surgical removal in our hospital; (III) surgical pathology confirmed meningioma based on the 2016 WHO classification [1]; (IV) assessed the tumour consistency by a unified standard. The exclusion criteria were as follows: (I) patients had undergone radiotherapy, chemotherapy, or surgery; and (II) insufficient quality of images. Finally, 52 patients pathologically confirmed as meningiomas were included in the studies. Of these, 2 patients were excluded due to a history of cranial radiotherapy, and 3 patients were excluded because of severe motion artefacts. The final cohort consisted of 47 patients. The patient flowchart is shown in Fig. 1.

### Intraoperative assessment of tumour consistency

The assessment was conducted by two senior neurosurgeons (with 15 and 10 years of experience, respectively) who performed the surgical resection together. Tumour consistency was evaluated according to Zada’s consistency grading system [26]. Both neurosurgeons agreed on the final grade of each tumour. Soft meningiomas were defined as those amenable to be removed totally or mainly with suction, which corresponded to Grade 1 and Grade 2 of Zada’s consistency grading system. Hard meningiomas were defined as those that required sharp resection, ultrasonic aspiration or with calcification the within tumour, which corresponded to Grade 3, Grade 4 ad Grade 5 of Zada’s consistency grading system [27].

### Image acquisition

MRI was performed using a 3 T MRI system (Discovery 750, GE Healthcare, Milwaukee, Wis, USA) equipped with an eight-channel receiver head coil. Conventional non-enhanced MRI sequences, multiple-b-value DWI, and contrast-enhanced T1-weigted imaging were performed in sequence. The total scan duration was 15 min 41 s.

Conventional non-enhanced MRI sequences included axial T1-weighted fluid attenuated inversion recovery (FLAIR) images (repetition time [TR] = 1,750 ms, echo time [TE] = 23 ms, section thickness = 5 mm, intersection gap = 1.5 mm, field of view [FOV] = 24 cm, matrix = 320 × 320); axial T2-weighted FSE images (TR = 6,488 ms, TE = 94 ms, section thickness = 5 mm, intersection gap = 1.5 mm, matrix = 512 × 512, FOV = 24 cm); axial T2-weighted FLAIR images (TR = 8,500 ms, TE = 143 ms, section thickness = 5 mm, intersection gap = 1.5 mm, matrix = 288 × 224, FOV = 24 cm).

DWI used a spin echo (SE)-echo planar imaging (EPI) diffusion sequence in the axial plane (TR = 5,000, TE = 84.6ms, section thickness = 5 mm, intersection gap = 0 mm, FOV = 24 cm, matrix = 192 × 192, number of sections = 30. Twelve b-values from 0 to 3,000 s/mm^2^ (0, 50, 100, 150, 200, 300, 500, 800, 1,000, 1,500, 2,000, and 3,000 s/mm^2^; with number of excitations [NEX] = 1 for b = 0-500 s/mm^2^, two NEX for b = 800-1,000 s/mm^2^, three NEX for b = 1,500 s/mm^2^, four NEX for b = 2000 s/mm^2^, and six NEX for b = 3000 s/mm^2 [22]^.

A contrast-enhanced three-dimensional (3D) axial T1-weighted fast spoiled gradient echo (FSPGR) was served as anatomical reference for DWI (TR = 8.2, TE = 3.2ms, section thickness = 1 mm, matrix = 256 × 256, FOV = 24 cm, inversion = 450ms, flip angle = 12°. Post-contrast images were obtained after administration of intravenous contrast material (0.1 mmol/kg, gadopentetate dimeglumine, Bayer Schering, Berlin, Germany) at a speed of 2 ml/s.

### MR image processing and histogram analysis

The obtained DWI data were transferred to a workstation (Advantage Workstation 4.6; GE Medical Systems) for further postprocessing. Parameter maps were generated through the MADC program in Functool software for each model.

The mono-exponential model was calculated with the following equation:

S(b)/S_0_ = exp (-b × ADC).

where S(b) is the mean signal intensity with diffusion gradient b, and S_0_ is the mean signal intensity without diffusion gradient [8]. In the present study, ADC was calculated from the mono-exponential model with two b-values (0, 1000 s/mm^2^).

The bi-exponential model was calculated with the following equation:

S(b)/S_0_ = [*f* × exp (-b × D*)] + [(1-*f*) ×exp (-b × D)]

where D is the pure molecular diffusion. D* is the pseudo-diffusion coefficient, and *f* is the microvascular volume fraction which represents the fraction of water diffusion relative to microcirculation [16].

The stretched-exponential model was calculated with the following equation:

S(b)/S0 = [exp (-b × DDC) ^alpha^]

where DDC is the distributed diffusion coefficient which reflects the mean intravoxel diffusion rate, and alpha indicates intravoxel diffusion heterogeneity ranging from 0 to 1 [18].

MEM data were linearly fitted with the least square method. BEM and SEM data were fitted by the LevenbergeMarquardt fit for non-linear fitting [28].

For each lesion, a three-dimensional volume of interest (VOI) of the whole tumour was semiautomatically delineated on all imaging slices with the ITK SNAP program (version 4.6.1, University of Pennsylvania, www.itksnap.org), by two independent radiologists (with 15 and 10 years of experience in neuroradiology, respectively). The VOIs of the solid tumour portion were delineated on axial contrast-enhanced T1WI by referring to conventional MR images. Necrosis, cystic portion, calcification and haemorrhage were carefully recognized and excluded from tumour portion. Then VOIs were automatically projected onto 6 diffusion maps (ADC, D, D*, *f*, DDC, alpha) by a co-registration tool in SPM8 (Wellcome Centre for Human Neuromaging, http://www.fl.ion.ucl.ac.uk/spm/).

The data of tumour solid parts were then assessed by an open-source software package FeAture Explorer (FAE; https://github.com/salan668/FAE). The 3D information from all voxels inside the VOIs was calculated to generate the histogram of 6 diffusion maps. The final extracted histogram parameters included mean, median, 10th percentile, 90th percentile, kurtosis, and skewness. The workflow chart is presented as Fig. S2.

### Statistical analysis

All data analysis was performed with IBM SPSS Statistics (Version 23.0, IBM Corp) and Medcalc (Version 11.1.1.0). The interobserver agreements of all histogram parameters were evaluated with Bland‒Altman analysis and the intraclass correlation coefficient (ICC): 0.00–0.20, poor correlation; 0.21–0.40, fair correlation; 0.41–0.60, moderate correlation; 0.61–0.80, good correlation; and 0.81–1.00, excellent correlation. Student’s t-test or nonparametric Mann‒Whitney U test was performed to test the differences in histogram parameters between soft and hard tumours. For statistically significant parameters, ROC curves were conducted to evaluate their diagnostic abilities in differentiating hard tumours from soft tumours. The corresponding area under the ROC curves (AUCs), with the 95% confidence interval (CI), was calculated to determine the optimal cut-off values for each histogram metric in the grading of meningioma. The diagnostic sensitivity and specificity of the value were also computed. The optimal threshold was selected by the maximum Youden index. The DeLong method was used to analyse the significance of the difference between the AUCs. P values less than 0.05 were considered statistically significant.

## Results

### Patient characteristics

A total of 47 patients were eligible for the study. Seventeen patients were in the hard meningioma group, while 30 were in the soft meningioma group. Patient characteristics are summarised in Table 1. There was no significant difference between soft and hard tumours on sex, age, histological grading or subtype.

**Table 1:** Patient characteristics of soft and hard meningiomas

	Soft	Hard	*p* Value
N	30	17	
Sex (% female)	23 (76.7)	11 (64.7)	0.378^a^
Age (years)	57 (25–79)	52 (38–71)	0.088^a^
Histopathological Grade (% high grade)	5 (16.7)	3 (17.6)	1^a^
Histopathological Subtype			0.424^b^
Meningothelial	7 (23.3)	4 (23.5)	
Fibroblastic	6 (20.0)	7 (41.2)	
Transitional	8 (26.7)	3 (17.6)	
Atypical	5 (16.7)	3 (17.6)	
Other subtypes	4 (13.3)	0 (0)	

Values are given as the mean (range) or n (%)

^a^Comparisons were performed by the Mann‒Whitney U test

^b^Comparisons were performed by χ2 test

### Interobserver agreement

As shown in Table S1, excellent inter-reader agreements were gained in the measurement of most histogram parameters except DDC kurtosis and skewness (fair and good agreement, respectively). Moreover, Bland–Altman analysis also showed good repeatability between the two observers and was considered clinically acceptable (Fig.S1).

### Comparisons of histogram parameters between soft and hard meningiomas

**Fig. 2:**
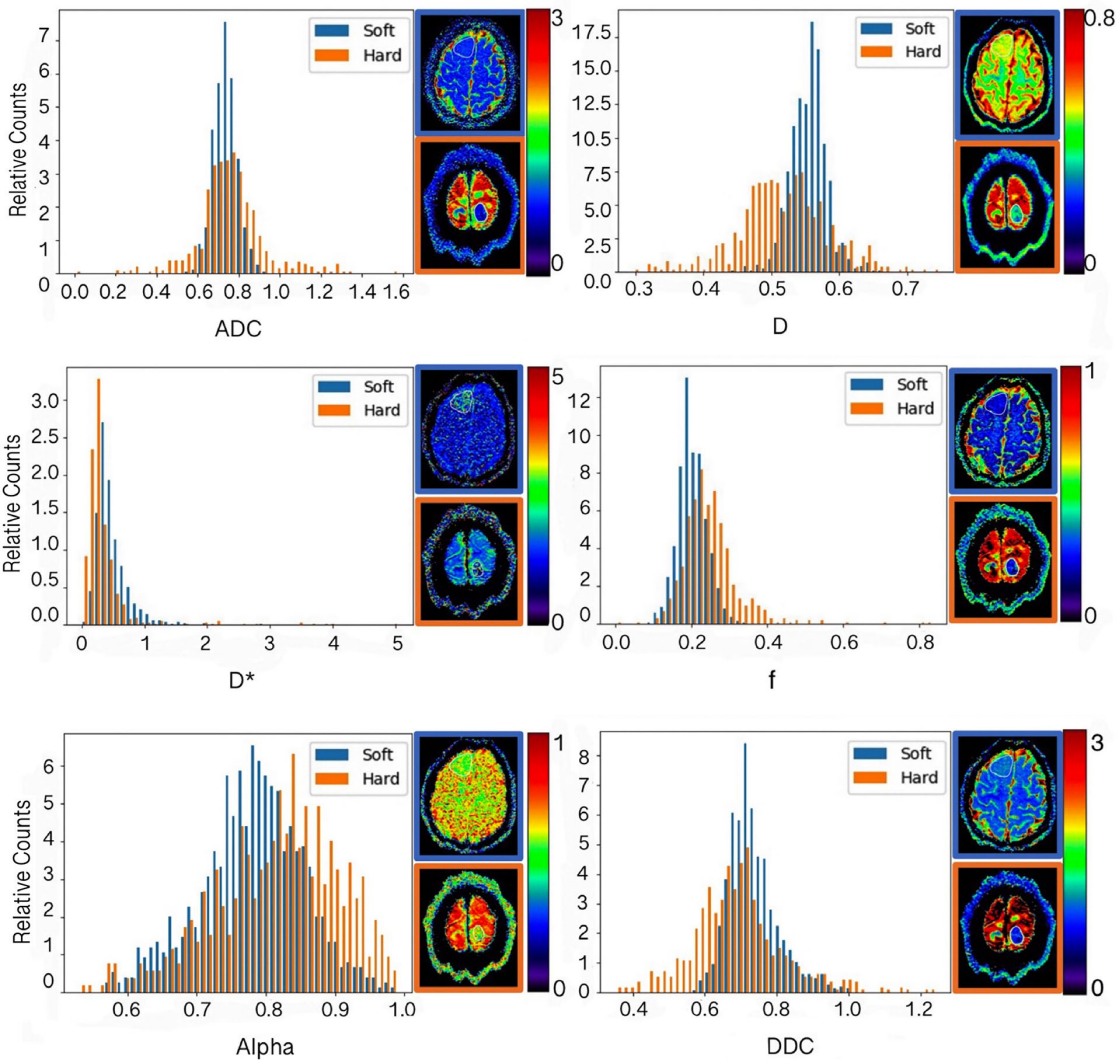
Histograms of ADC, alpha, DDC, fast-ADC, fraction of fast-ADC and slow-ADC from a soft meningioma in a 48-year-old woman (blue) and a hard meningioma in a 54-year-old woman (orange). The corresponding diffusion maps are on the right side of each histogram (the soft tumour with a blue border, the hard tumour with an orange border)

The results of comparison of all histogram parameters between hard and soft meningiomas are presented in Table 2. The 10th percentile of DDC, D*, and ADC were significantly lower in hard tumours than in soft tumours (P ≤ 0.05). The alpha 90th percentile was significantly higher in hard tumours than in soft tumours (P < 0.02). No significant difference was found for other parameters between the two groups. Metric maps and histograms of two representative subjects are presented in Fig. 2.

**Table 2:** Comparison of histogram parameters of diffusion metrics between soft and hard meningiomas

Histogram Parameters	Tumour Consistency		*p* Value
	Soft	Hard	
ADC (×10^− 3^ mm^2^/sec)			
10th percentile	0.69 ± 0.09	0.60 ± 0.26	**0.044** ^**b**^ *
90th percentile	0.91 ± 0.14	0.92 ± 0.42	0.324^b^
Mean	0.82 ± 0.11	0.80 ± 0.22	0.982^b^
Median	0.81 ± 0.11	0.78 ± 0.20	0.626^b^
Kurtosis	7.19 ± 6.77	7.95 ± 4.88	0.492^b^
Skewness	0.73 ± 0.95	0.97 ± 0.97	0.929^b^
D (×10^− 3^ mm^2^/sec)			
10th percentile	0.49 ± 0.05	0.43 ± 0.22	0.054^b^
90th percentile	0.61 ± 0.06	0.63 ± 0.13	0.358^b^
Mean	0.55 ± 0.05	0.52 ± 0.12	0.432^a^
Median	0.55 ± 0.05	0.55 ± 0.14	0.479^b^
Kurtosis	5.13 ± 6.28	5.25 ± 6.15	0.808^b^
Skewness	0.40 ± 0.89	0.35 ± 1.17	0.808^b^
D* (×10^− 3^ mm^2^/sec)			
10th percentile	2.38 ± 0.77	1.64 ± 1.64	**< 0.001** ^**b**^ **
90th percentile	6.17 ± 4.09	6.97 ± 5.11	0.288^b^
Mean	4.20 ± 2.09	4.22 ± 2.15	0.859^b^
Median	3.74 ± 1.85	3.52 ± 1.10	0.104^b^
Kurtosis	38.08 ± 129.75	46.76 ± 54.62	0.674^b^
Skewness	4.22 ± 5.16	4.71 ± 3.04	0.400^b^
*F*			
10th percentile	0.20 ± 0.04	0.19 ± 0.08	0.278^b^
90th percentile	0.32 ± 0.08	0.33 ± 0.11	0.501^b^
Mean	0.25 ± 0.06	0.28 ± 0.12	0.232^b^
Median	0.25 ± 0.04	0.25 ± 0.10	0.912^b^
Kurtosis	23.60 ± 40.09	7.06 ± 22.71	0.057^b^
Skewness	2.92 ± 1.78	2.43 ± 2.07	0.391^a^
Alpha			
10th percentile	0.69 ± 0.09	0.64 ± 0.12	0.215^b^
90th percentile	0.85 ± 0.05	0.93 ± 0.05	**< 0.001** ^**a**^ **
Mean	0.77 ± 0.07	0.78 ± 0.07	0.084^b^
Median	0.77 ± 0.07	0.80 ± 0.07	0.068^b^
Kurtosis	3.90 ± 0.80	3.13 ± 1.46	0.073^b^
Skewness	0.09 ± 0.47	0.31 ± 0.69	0.068^b^
DDC (×10^− 3^ mm^2^/sec)			
10th percentile	0.68 ± 0.11	0.58 ± 0.37	**0.020** ^**b**^ *
90th percentile	0.91 ± 0.20	0.93 ± 0.58	0.191^b^
Mean	0.78 ± 0.17	0.78 ± 0.25	0.965^b^
Median	0.77 ± 0.16	0.72 ± 0.16	0.113^b^
Kurtosis	9.43 ± 13.35	11.29 ± 46.94	0.207^b^
Skewness	1.19 ± 2.33	1.78 ± 4.63	0.199^b^

Data are presented as the mean ± standard deviation (normalized distribution) or median ± interquartile range (skewness distribution) ADC, D, D*, and DDC are in units of ×10^− 3^ mm^2^/sec

**p < 0.01, *p < 0.05

^a^ Comparisons were performed by independent samples t-test

^b^ Comparisons were performed by Mann‒Whitney U test

### Diagnostic performances of histogram parameters in distinguishing soft and hard meningiomas

**Fig. 3:**
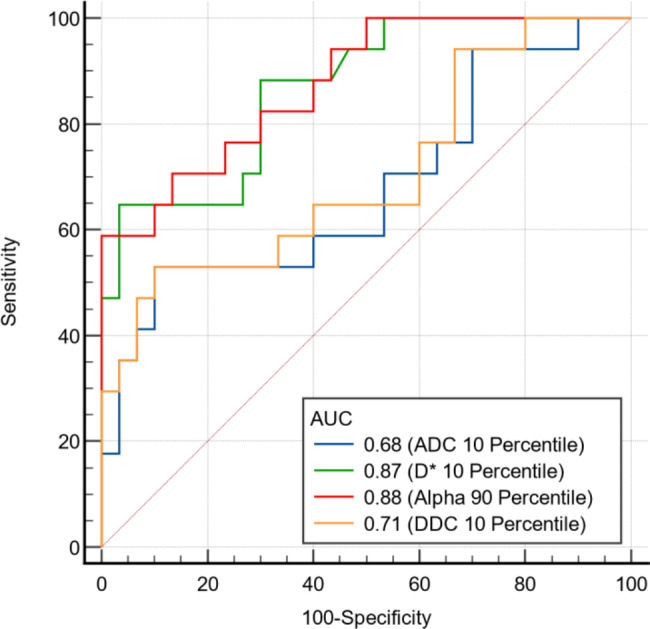
ROC curves of histogram parameters in differentiating soft and hard meningiomas

The results of ROC analyses of the significant histogram parameters are presented in Table 3; Fig. 3. The alpha 90th percentile yielded the highest AUC of 0.88. The optimal cut-off value of alpha was 0.91 with a sensitivity of 58.52%, a specificity of 100.00% and an accuracy of 85.10% in the diagnosis of hard meningiomas. The D* 10th percentile had a relatively high AUC value of 0.86. When differentiating two groups by the optimal cut-off value of 17.30, a sensitivity of 64.71%, a specificity of 96.67% and an accuracy of 83.00% were obtained in diagnosing hard meningiomas. The ADC 10th percentile and DDC 10th percentile had relatively lower AUCs (0.68 and 0.71, respectively). The Delong test demonstrated that alpha 90th percentile had a significantly greater AUC value than the ADC 10th percentile (P ≤ 0.05). The D* 10th percentile had a significantly greater AUC value than ADC 10th percentile and DDC 10th percentile (P ≤ 0.03). No significant difference between the alpha 90th percentile and D* 10th percentile or between the alpha 90th percentile and DDC 10th percentile was observed.

**Table 3:** Diagnostic performances of histogram parameters in differentiating hard from soft meningiomas

Histogram Parameters	AUC (95%CI)	Cut-off	Se (%)	Sp (%)	Youden Index	Accuracy (%)	PPV (%)	NPV (%)
ADC 10th percentile	0.68 (0.53, 0.81)	0.60	52.94	90.00	0.43	76.60	75.00	77.14
D* 10th percentile	0.87 (0.77, 0.95)	17.30	64.71	96.67	0.61	83.00	91.67	82.86
Alpha 90th percentile	0.88 (0.75, 0.95)	0.91	58.82	100.00	0.59	85.10	100.00	75.00
DDC 10th percentile	0.71 (0.56, 0.83)	0.58	52.94	90.00	0.43	76.60	90.91	80.56

Note: Se represents sensitivity. Sp represents specificity. PPV represents positive predictive value. NPV represents negative predictive value

## Discussion

Preoperative prediction of meningioma consistency is of vital clinical importance. DWI is a promising approach for evaluating the tumour biology [8]. In this study, MEM, BEN, and SEM DWI were applied to preoperatively predict the consistency of meningiomas. The present results demonstrated that the differentiation between hard and soft meningiomas is feasible through different models of DWI with histogram analysis. D* (BEM-DWI) and alpha (SEM-DWI) showed significantly better diagnostic performances than ADC (MEM-DWI) Moreover, among all histogram parameters, only percentile values (10th and 90th percentiles) showed significant differences between soft and hard tumours.

In the current study, the ADC 10th percentile derived from MEM was significantly lower in hard meningiomas than in soft tumours. Our result is consistent with several previous studies on meningiomas [10, 11] and pituitary adenomas [29], which are the most explored intracranial tumours in preoperative tumour consistency prediction. However, there were also a few incoherent results. Phuttharak et al. found that hard meningiomas had higher ADC values and ADC ratios than soft tumours [12]. Watanabe et al. indicated that there was no correlation between meningioma consistency and the ADC value. Despite the various methodologies in these studies [29], these controversial conclusions may also be explained from a histopathological perspective. Harder tumour consistency is considered to be related to higher tumour cellularity with stronger cell adhesion [3], more fibrous components [4, 5] and lower vascularity levels [5–7]. Meanwhile, a lower ADC value was suggested to be associated with a higher cell density and fibrous components [29, 30]. However, ADC reflects the overall diffusion level within a voxel and cannot separately present different diffusion patterns [15]. Hence, we cannot distinguish different histopathological components based on ADC values, which may underlie the mismatch between them. Miyoshi et al. indicated that the increased cellularity with stronger adhesion accounted for the lower ADC value in hard meningiomas [10]. While Romano et al. held the opposite view that hard specimens from pituitary adenomas had low cellularity and a high percentage of collagen content [5] which may indicate that the decreased ADC value may be due to the collagen content rather than the high cellularity. Several advanced diffusion models have been developed to generate parameters that reflect more specific diffusion patterns. To the best of our knowledge, the current study is the first study to apply BEM and SEM to predict tumour consistency. Notably, magnetic resonance elastography (MRE) seems to be able to bypass the histological complexity to provide tumour stiffness information by applying a mechanical wave [31, 32]. However, this technology requires specialized hardware that is not widely used in clinical practice [31, 32].In addition, it remains unknown whether mechanical vibration harms the brain [10]. Thus, Le Bihan et al. used a virtual MRE approach to yield a novel metric named shift ADC (b = 200, 1500s/mm^2^). However, it failed to predict meningioma [10] and pituitary adenoma [33] consistency evaluated during surgery.

In the present study, the D* 10th percentile was significantly lower in the hard meningiomas with an excellent AUC of 0.86. This result indicated that the hypervascular tumour tended to be softer, which was consistent with previous studies. Romano et al. [5] found that specimens from soft components of pituitary adenomas were characterized by the high representation of vascularization and microhemorrhage, and the signal intensity ratio value obtained by dynamic contrast-enhanced T1WI could distinguish soft and hard components. Phuttharak et al. found that meningiomas with hard consistency had a significant correlation with the absence of a vascular core [12]. Takamura et al. reported that the relative mean transit time (MTT) measured by CT perfusion was inversely correlated with stiffness measured by MRE [6]. Flagstad et al. also suggested that decreased tissue stiffness in glioblastoma was associated with increased cerebral blood flow [7]. In the present study, none of the histogram parameters of D or *f* were significantly different between soft and hard tumours. This may indicate that D* is a more sensitive biomarker than *f* when predicting meningioma consistency. The insignificant result of D demonstrates that the perfusion-free diffusion level does not correlate with tumour consistency. Moreover, the significant difference in the ADC 10th percentile may be ascribable to the perfusion component inside the ADC value. Thus, we speculated that perfusion or vascularity contributes more to tumour consistency than cellularity.

In the present study, the DDC 10th percentile was significantly lower in hard tumours, which is in line with our result of ADC value results. In a previous study [22], a lower DDC value was correlated with tumour aggressiveness, which usually demonstrated higher mitotic activity, necrosis, nuclear atypia, and small cells with increased intracellular complex protein molecules and nucleus to cytoplasm ratio [34]. Although stronger intercellular adhesion and higher cellular density may be an explanation [3, 10], these pathological characteristics can induce firmer consistency and lower DDC value of tumours still needs further investigation. The alpha 90th percentile in the present study was significantly higher in hard tumours and yielded the highest AUC of 0.88. This result demonstrates a lower degree of multiexponential decay of signal [18] in hard meningiomas than in soft meningiomas. Hyung et al. reported a significantly higher alpha value in hypovascular focal liver lesions than in hypervascular lesions [19]. Orton et al. found that alpha value significantly increased after treatment with vascular endothelial growth factor inhibitors [35]. These two studies both proved that the alpha value is highly impacted by the vascular portion in tissue. Notably, this viewpoint is in line with the result of D* that harder tumours have lower D* indicating less vascular structure or lower microperfusion. Thus, the excellent performance of alpha and D* in differentiating hard from soft tumours might be explained through the reflection of tissue vascular portion or microperfusion. Therefore, radiologists and neurosurgeons should pay more attention to meningiomas showing lower D* or higher alpha, as the tumour is likely to be harder. A stricter pre- and intra-operative management is needed. Interestingly, neither kurtosis nor skewness parameters turned out to have significant differences between hard and soft tumours in the present study. These results show the diverse feasibilities of intravoxel and intervoxel level metrics. Since alpha, the intravoxel metric, had a better performance of differentiation between hard and soft meningiomas, we suggest studies in the future focusing more on the intravoxel scale to explore tumour consistency.

In this study, only the histogram percentile parameters (10th and 90th percentiles) were proven to be feasible to predict meningioma consistency. While the conventional parameters (mean and median) failed, which is consistent with previous studies [25, 36]. Our results suggest that histogram parameters can act as more promising biomarkers than conventional metrics in revealing tumour microstructure, especially intratumuoral heterogeneity.

This study still has several limitations. First, the sample size was relatively small. A larger and multi-centre study population may further verify the present findings. Second, the classification of tumour consistency was still based on a subjective method. Several studies used durometers to measure the consistency of tumours [10, 32]. Third, the association of diffusion parameters with pathological characteristics was not performed in this study. Detailed histological parameters, such as cell density, fibrous content and vascularity need to be explored in the future to demonstrate the associations.

## Conclusion

Different models of DWI, including MEM, BEM, and SEM, are useful in the differentiation between soft and hard meningiomas. Moreover, histogram percentile parameters (10th, 90th percentile) outperform conventional parameters such as the mean or median in distinguishing between two groups. Alpha and D* have significantly better diagnostic performances than ADC and DDC in identifying hard meningiomas.

### Electronic supplementary material

Below is the link to the electronic supplementary material.

Supplementary Material 1

Supplementary Material 2

Supplementary Material 3

Supplementary Material 4

## Data Availability

All data generated or during this study are included in this published article and the supplementary materials. Other data that are relevant to this article are available from the corresponding authors upon reasonable request.

## Abbreviations

MRI: Magnetic resonance imaging
DWI: Diffusion-weighted imaging
MEM: Mono-exponential model
BEM: Bi-exponential model
SEM: Stretched exponential model
ADC: Apparent diffusion coefficient
D: Pure molecular diffusion
D*: Pseudo-diffusion coefficient
f: Perfusion fraction
DDC: Distributed diffusion coefficient
Alpha: Intravoxel diffusion heterogeneity
AUC: Area under the ROC curves
CI: Confidence interval
ICC: Intraclass correlation coefficient
MTT: Mean transit time

## References

[1] Louis DN, Perry A, Reifenberger G, von Deimling A, Figarella-Branger D, Cavenee WK, Ohgaki H, Wiestler OD, Kleihues P, Ellison DW (2016). The 2016 World Health Organization Classification of Tumors of the Central Nervous System: a summary. Acta Neuropathol.

[2] Itamura K, Chang K-E, Lucas J, Donoho DA, Giannotta S, Zada G. Prospective clinical validation of a meningioma consistency grading scheme: association with surgical outcomes and extent of Tumor resection. J Neurosurg. 2018;1–5. 10.3171/2018.7.JNS1838 30554187

[3] Muthupillai R, Rossman PJ, Lomas DJ, Greenleaf JF, Riederer SJ, Ehman RL (1996). Magnetic resonance imaging of transverse acoustic strain waves. Magn Reson Med.

[4] Mahmoud OM, Tominaga A, Amatya VJ, Ohtaki M, Sugiyama K, Sakoguchi T, Kinoshita Y, Takeshima Y, Abe N, Akiyama Y (2011). Role of PROPELLER diffusion-weighted imaging and apparent diffusion coefficient in the evaluation of pituitary adenomas. Eur J Radiol.

[5] Romano A, Coppola V, Lombardi M, Lavorato L, Di Stefano D, Caroli E, Rossi Espagnet MC, Tavanti F, Minniti G, Trillò G, Bozzao A (2017). Predictive role of dynamic contrast enhanced T1-weighted MR sequences in pre-surgical evaluation of macroadenomas consistency. Pituitary.

[6] Takamura T, Motosugi U, Ogiwara M, Sasaki Y, Glaser KJ, Ehman RL, Kinouchi H, Onishi H (2021). Relationship between Shear Stiffness measured by MR Elastography and Perfusion Metrics measured by Perfusion CT of Meningiomas. AJNR Am J Neuroradiol.

[7] Fløgstad Svensson S, Fuster-Garcia E, Latysheva A, Fraser-Green J, Nordhøy W, Isam Darwish O, Thokle Hovden I, Holm S, Vik-Mo EO, Sinkus R, Eeg Emblem K (2022). Decreased tissue stiffness in glioblastoma by MR Elastography is associated with increased cerebral blood flow. Eur J Radiol.

[8] Le Bihan D (1995). Molecular diffusion, tissue microdynamics and microstructure. NMR Biomed.

[9] Lin L, Chen X, Jiang R, Zhong T, Du X, Xu G, Duan Q, Xue Y (2018). Differentiation between vestibular schwannomas and meningiomas with atypical appearance using diffusion kurtosis imaging and three-dimensional arterial spin labeling imaging. Eur J Radiol.

[10] Miyoshi K, Wada T, Uwano I, Sasaki M, Saura H, Fujiwara S, Takahashi F, Tsushima E, Ogasawara K. Predicting the consistency of intracranial meningiomas using apparent diffusion coefficient maps derived from preoperative diffusion-weighted imaging. J Neurosurg. 2020;1–8. 10.3171/2020.6.JNS20740 33186907

[11] Yogi A, Koga T, Azama K, Higa D, Ogawa K, Watanabe T, Ishiuchi S, Murayama S (2014). Usefulness of the apparent diffusion coefficient (ADC) for predicting the consistency of intracranial meningiomas. Clin Imaging.

[12] Phuttharak W, Boonrod A, Thammaroj J, Kitkhuandee A, Waraasawapati S (2018). Preoperative MRI evaluation of meningioma consistency: a focus on detailed architectures. Clin Neurol Neurosurg.

[13] Watanabe K, Kakeda S, Yamamoto J, Ide S, Ohnari N, Nishizawa S, Korogi Y (2016). Prediction of hard meningiomas: quantitative evaluation based on the magnetic resonance signal intensity. Acta Radiol (Stockholm Sweden: 1987).

[14] Alyamany M, Alshardan MM, Jamea AA, ElBakry N, Soualmi L, Orz Y (2018). Meningioma consistency: correlation between magnetic resonance imaging characteristics, operative findings, and histopathological features. Asian J Neurosurg.

[15] Filippi CG, Edgar MA, Uluğ AM, Prowda JC, Heier LA, Zimmerman RD (2001). Appearance of meningiomas on diffusion-weighted images: correlating diffusion constants with histopathologic findings. AJNR Am J Neuroradiol.

[16] Le Bihan D, Breton E, Lallemand D, Aubin ML, Vignaud J, Laval-Jeantet M (1988). Separation of diffusion and perfusion in intravoxel incoherent motion MR imaging. Radiology.

[17] Mulkern RV, Gudbjartsson H, Westin CF, Zengingonul HP, Gartner W, Guttmann CR, Robertson RL, Kyriakos W, Schwartz R, Holtzman D (1999). Multi-component apparent diffusion coefficients in human brain. NMR Biomed.

[18] Bennett KM, Schmainda KM, Bennett RT, Rowe DB, Lu H, Hyde JS (2003). Characterization of continuously distributed cortical water diffusion rates with a stretched-exponential model. Magn Reson Med.

[19] Kim HC, Seo N, Chung YE, Park M-S, Choi J-Y, Kim M-J (2019). Characterization of focal liver lesions using the stretched exponential model. Comparison with monoexponential and biexponential diffusion-weighted magnetic resonance imaging. Eur Radiol.

[20] Kusunoki M, Kikuchi K, Togao O, Yamashita K, Momosaka D, Kikuchi Y, Kuga D, Hata N, Mizoguchi M, Iihara K (2020). Differentiation of high-grade from low-grade diffuse gliomas using diffusion-weighted imaging. A comparative study of mono-, bi-, and stretched-exponential diffusion models. Neuroradiology.

[21] Jin Y-N, Zhang Y, Cheng J-L, Zheng D-D, Hu Y, Monoexponential (2019). Biexponential, and stretched-exponential models using diffusion-weighted imaging: a quantitative differentiation of breast lesions at 3.0T. J Magn Reson Imaging: JMRI.

[22] Lin L, Xue Y, Duan Q, Chen X, Chen H, Jiang R, Zhong T, Xu G, Geng D, Zhang J (2019). Grading meningiomas using mono-exponential, bi-exponential and stretched exponential model-based diffusion-weighted MR imaging. Clin Radiol.

[23] Keil VC, Mädler B, Gielen GH, Pintea B, Hiththetiya K, Gaspranova AR, Gieseke J, Simon M, Schild HH, Hadizadeh DR (2017). Intravoxel incoherent motion MRI in the brain: impact of the fitting model on perfusion fraction and lesion differentiability. J Magn Reson Imaging: JMRI.

[24] Togao O, Hiwatashi A, Yamashita K, Kikuchi K, Momosaka D, Yoshimoto K, Kuga D, Mizoguchi M, Suzuki SO, Iwaki T (2018). Measurement of the perfusion fraction in brain tumors with intravoxel incoherent motion MR imaging: validation with histopathological vascular density in meningiomas. Br J Radiol.

[25] Just N (2014). Improving tumour heterogeneity MRI assessment with histograms. Br J Cancer.

[26] Zada G, Yashar P, Robison A, Winer J, Khalessi A, Mack WJ, Giannotta SL (2013). A proposed grading system for standardizing Tumor consistency of intracranial meningiomas. NeuroSurg Focus.

[27] Zhai Y, Song D, Yang F, Wang Y, Jia X, Wei S, Mao W, Xue Y, Wei X (2021). Preoperative prediction of Meningioma consistency via machine learning-based Radiomics. Front Oncol.

[28] Xiao Z, Tang Z, Qiang J, Wang S, Qian W, Zhong Y, Wang R, Wang J, Wu L, Tang W, Zhang Z (2018). Intravoxel Incoherent Motion MR Imaging in the differentiation of Benign and Malignant Sinonasal lesions: comparison with Conventional Diffusion-Weighted MR Imaging. AJNR Am J Neuroradiol.

[29] Ding W, Huang Z, Zhou G, Li L, Zhang M, Li Z (2021). Diffusion-weighted imaging for predicting Tumor consistency and extent of resection in patients with pituitary adenoma. Neurosurg Rev.

[30] Boxerman JL, Rogg JM, Donahue JE, Machan JT, Goldman MA, Doberstein CE (2010). Preoperative MRI evaluation of pituitary macroadenoma: imaging features predictive of successful transsphenoidal Surgery. AJR Am J Roentgenol.

[31] Murphy MC, Huston J, Glaser KJ, Manduca A, Meyer FB, Lanzino G, Morris JM, Felmlee JP, Ehman RL (2013). Preoperative assessment of meningioma stiffness using magnetic resonance elastography. J Neurosurg.

[32] Hughes JD, Fattahi N, Van Gompel J, Arani A, Meyer F, Lanzino G, Link MJ, Ehman R, Huston J. Higher-resolution magnetic resonance elastography in Meningiomas to Determine Intratumoral consistency. Neurosurgery. 2015;77. 10.1227/NEU.0000000000000892 PMC4749919 26197204

[33] Lagerstrand K, Gaedes N, Eriksson S, Farahmand D, De Coursey E, Johansson G, Jönsson L, Skoglund T (2021). Virtual magnetic resonance elastography has the feasibility to evaluate preoperative pituitary adenoma consistency. Pituitary.

[34] Nagar VA, Ye JR, Ng WH, Chan YH, Hui F, Lee CK, Lim CCT (2008). Diffusion-weighted MR imaging: diagnosing atypical or malignant meningiomas and detecting Tumor dedifferentiation. AJNR Am J Neuroradiol.

[35] Orton MR, Messiou C, Collins D, Morgan VA, Tessier J, Young H, deSouza N, Leach MO (2016). Diffusion-weighted MR imaging of metastatic abdominal and pelvic tumours is sensitive to early changes induced by a VEGF inhibitor using alternative diffusion attenuation models. Eur Radiol.

[36] Cao T, Jiang R, Zheng L, Zhang R, Chen X, Wang Z, Jiang P, Chen Y, Zhong T, Chen H (2023). T1 and ADC histogram parameters may be an in vivo biomarker for predicting the grade, subtype, and proliferative activity of meningioma. Eur Radiol.

