# Supplementary material for: Histogram analysis of mono-exponential, bi-exponential and stretched-exponential diffusion-weighted MR imaging in predicting consistency of meningiomas
Source: Cancer Imaging. 2023 Dec 5;23:117. doi: 10.1186/s40644-023-00633-z (PMC10696773; doi:10.1186/s40644-023-00633-z)
Supplement: Supplementary file 3 — Supplementary Material 3 [file 40644_2023_633_MOESM3_ESM.docx]

**The following changes have been made in response to the comments of Reviewer #1:**

1. Authors have addressed the issues I proposed. this revised manuscript is easy to follow.

Response:

We earnestly appreciate the positive comment from the reviewer.

**The following changes have been made in response to the comments of Reviewer #2:**

1. The authors almost resolved my concerns for the previous version. However, a few comments can be considered.

Response:

Thank the reviewer for carefully reading our manuscript. We are grateful for the reviewer’s affirmation of our study.

1. The Conclusion of Abstract may revise as follows: "Histogram parameters of Alpha and D* may serve as better imaging biomarkers to aid in predicting the consistency of meningioma".

Response:

We appreciate the reviewer for the suggestion. We have revised the Conclusion of Abstract in the manuscript strictly as the reviewer suggested.

1. Line 36, Page 7: Please consider to revise "minimal damage" to "minimal invasion".

Response:

Thank the reviewer for his/her excellent suggestion. We totally agree that “minimal invasion” is more appropriate expression. We have revised the manuscript content.

4. Why did the authors only choose histogram parameters of mean, median, 10th percentile, 90th percentile, kurtosis, and skewness? Please explain. In addition, please present the workflow of the histogram analysis.

Response:

Thank the reviewer for remind us of such important issue.

As we all know, histogram analysis approach is mainly introduced to improve the assessment of tumour heterogeneity [1]. Percentile measurements, kurtosis and skewness are classic and mostly common used histogram parameters that can act as biomarkers of tumour heterogeneity [2]. Back in 2001, J. Dehmeshki et al [3] studied multiple sclerosis by histogram parameters (histogram peak height, peak location, average value, and 25th, 50th, and 75th centile values). Although histogram peak height and peak location are not exactly same to kurtosis and skewness, respectively, their values all depend on the peak of histogram. This shape analysis approach of histogram is essentially different from traditional methods. And the reason of choosing 10th and 90th percentiles instead of other percentiles is because the software (FeAture Explorer V.0.5.3) we used to extract histogram parameters can only provide these two percentiles, which are also the parameters recommended by the Imaging Biomarker Standardization Initiative (IBSI). In addition, Jan Brabec et al [4] found that lower 10th percentiles of DKI metrics in the whole-tumour were associated with hard meningiomas consistency. Their study also inspired us to predict meningioma consistency by percentile measurement. At present, increasing number of histogram parameters are studied [5], while the ability of the novel histogram parameters to provide additional physiological information remains controversial. For example, Root mean square is the square-root of the mean of all the squared intensity values. It can be regarded as another form of Mean so that it is likely to provide similar physiological information as Mean. Moreover, a more complicated mathematical process may further make it less explicable. Finally, we included mean and median as traditional metrics to explore whether advanced histogram parameters could obtain better diagnostic performance.

Meanwhile, we agree that including other histogram parameters can make our study more comprehensive. Then we additionally analyzed twelve histogram parameters of six diffusion metrics. Together with parameters we studied previously, the total number of histogram parameters is one hundred and eight. There were 52 parameters gaining significant difference between soft and hard meningiomas. While none of them have obtained a better AUC value than alpha 90th percentile or D* 10th percentile. The definitions of histogram parameters and the AUCs of 52 significant parameters were presented as below.

Besides, the workflow of histogram analysis has been added into Supplementary materials and presented as below as well.

References

[1] Xie T , Zhao Q , Fu C ,et al. Improved value of whole-lesion histogram analysis on DCE parametric maps for diagnosing small breast cancer (≤1cm)[J].European Radiology:1-10.DOI:10.1007/s00330-021-08244-7.

[2] Just,N. Improving tumour heterogeneity MRI assessment with histograms.[J].British Journal of Cancer, 2014.DOI:10.1038/bjc.2014.512.

[3] Dehmeshki J , Ruto A C , Arridge S ,et al. Analysis of MTR histograms in multiple sclerosis using principal components and multiple discriminant analysis[J].Magnetic Resonance in Medicine Official Journal of the Society of Magnetic Resonance in Medicine, 2001, 46(3):600.DOI:10.1002/mrm.1233.

[4] Brabec J , Szczepankiewicz F , Lennartsson F ,et al. Histogram analysis of tensor-valued diffusion MRI in meningiomas: Relation to consistency, histological grade and type[J].NeuroImage. Clinical, 2022, 33:102912.DOI:10.1016/j.nicl.2021.102912.

[5] Dejun S , Hao H , Wei G ,et al. Grading meningiomas with diffusion metrics: a comparison between diffusion kurtosis, mean apparent propagator, neurite orientation dispersion and density, and diffusion tensor imaging[J].European radiology. 2023.DOI:10.1007/s00330-023-09505-3.

| Table.1 The histogram parameters definitions | |
| --- | --- |
| First order features | Definitions |
| 10th percentile | The 10^th^ percentile of intensity values within the VOI |
| 90th percentile | The 90^th^ percentile of intensity values within the VOI |
| Minimum | The minimum of intensity values within the VOI |
| Maximum | The maximum of intensity values within the VOI |
| Mean | The average of intensity values within the VOI |
| Median | The median of intensity values within the VOI |
| Energy | A measure of the magnitude of voxel values |
| Entropy | The uncertainty/randomness in the image values |
| Interquartile Range | The range of 25^th^ and 75^th^ percentile of intensity values |
| Kurtosis | A measure of the peakedness of the distribution of values |
| Mean Absolute Deviation | The mean distance of all intensity values from the Mean Value of the image array |
| Range | The range of intensity values |
| Robust Mean Absolute Deviation | The mean distance of all intensity values from the Mean Value calculated on the subset of image array with gray levels in between, or equal to the 10th and 90th percentile. |
| Root Mean Squared | The square-root of the mean of all the squared intensity values |
| Skewness | The asymmetry of the distribution of values about the Mean value |
| Total Energy | The value of Energy feature scaled by the volume of voxel |
| Uniformity | A measure of the sum of the squares of each intensity value |
| Variance | The mean of the squared distances of each intensity value from the Mean value |

| Table. 2 AUCs of significant histogram parameters. | |
| --- | --- |
| Histogram parameter | AUC value |
| Alpha 90th Percentile | 0.876 |
| D* 10th Percentile | 0.868 |
| Alpha Variance | 0.855 |
| Alpha Mean Absolute Deviation | 0.851 |
| Alpha Interquartile Range | 0.839 |
| Alpha Robust Mean Absolute Deviation | 0.837 |
| f Variance | 0.829 |
| f Mean Absolute Deviation | 0.808 |
| Alpha Entropy | 0.787 |
| Alpha Range | 0.776 |
| Alpha Uniformity | 0.776 |
| Alpha Minimum | 0.766 |
| f Interquartile Range | 0.755 |
| f Robust Mean Absolute Deviation | 0.753 |
| D Interquartile Range | 0.748 |
| D* Variance | 0.743 |
| DDC Variance | 0.741 |
| DDC Range | 0.739 |
| D Mean Absolute Deviation | 0.739 |
| D Robust Mean Absolute Deviation | 0.739 |
| f Minimum | 0.738 |
| ADC Variance | 0.735 |
| D Variance | 0.735 |
| ADC Minimum | 0.729 |
| ADC Entropy | 0.725 |
| D Entropy | 0.725 |
| DDC Maximum | 0.724 |
| ADC Mean Absolute Deviation | 0.722 |
| D* Maximum | 0.722 |
| D* Mean Absolute Deviation | 0.722 |
| D Minimum | 0.722 |
| D* Range | 0.720 |
| f Range | 0.720 |
| D Range | 0.714 |
| ADC Interquartile Range | 0.712 |
| ADC Uniformity | 0.712 |
| ADC Range | 0.710 |
| ADC Robust Mean Absolute Deviation | 0.710 |
| DDC Minimum | 0.710 |
| f Root Mean Squared | 0.710 |
| DDC Mean Absolute Deviation | 0.706 |
| DDC 10th Percentile | 0.705 |
| D* Robust Mean Absolute Deviation | 0.704 |
| f Entropy | 0.702 |
| Alpha Root Mean Squared | 0.690 |
| f Energy | 0.688 |
| f Total Energy | 0.688 |
| D* Interquartile Range | 0.681 |
| ADC 10th Percentile | 0.678 |
| DDC Entropy | 0.673 |
| D Uniformity | 0.665 |


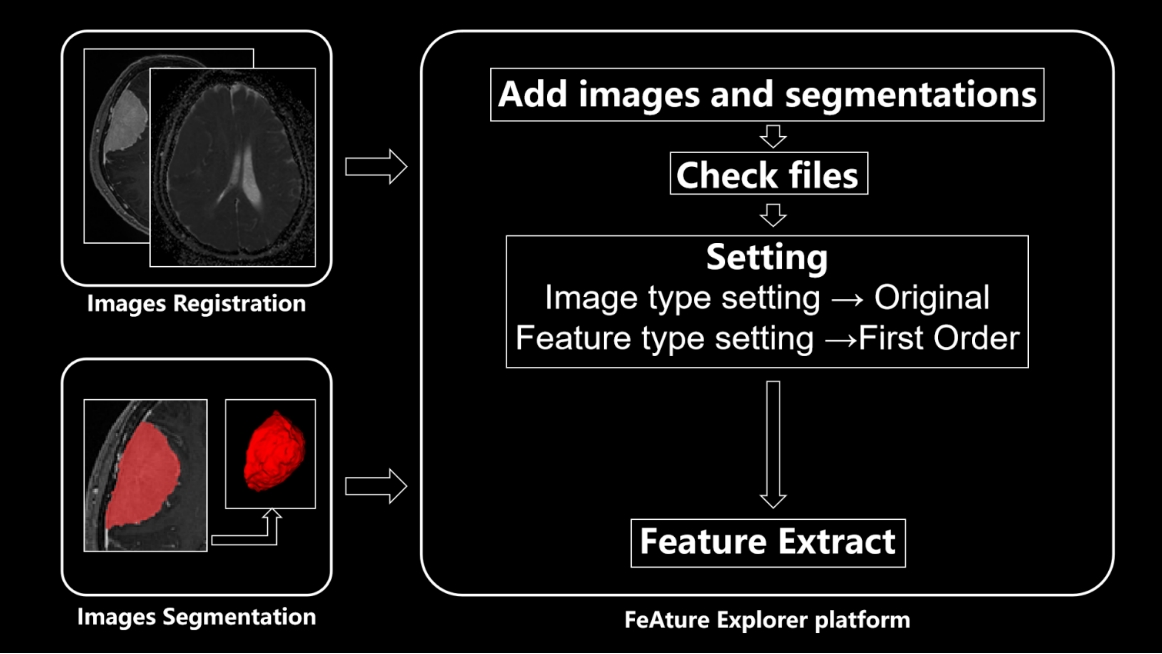


Figure S2. Workflow chart of histogram analysis
