# Supplementary material for: Histogram analysis of mono-exponential, bi-exponential and stretched-exponential diffusion-weighted MR imaging in predicting consistency of meningiomas
Source: Cancer Imaging. 2023 Dec 5;23:117. doi: 10.1186/s40644-023-00633-z (PMC10696773; doi:10.1186/s40644-023-00633-z)
Supplement: Supplementary file 4 — Supplementary Material 4 [file 40644_2023_633_MOESM4_ESM.docx]

| **Supplementary Table 1.** Inter-observer variability of histogram parameters of meningiomas | |
| --- | --- |
| **Histogram Parameters** | **Intra-class correlation coefficient**  **(95% CI for inter-observer)** |
| ADC |  |
| 10th percentile | 0.993 (0.978-0.998) |
| 90th percentile | 0.909 (0.746-0.970) |
| Mean | 0.995 (0.986-0.999) |
| Median | 0.982 (0.946-0.994) |
| Kurtosis | 0.879 (0.669-0.959) |
| Skewness | 0.922 (0.775-0.974) |
| D |  |
| 10th percentile | 0.984 (0.952-0.995) |
| 90th percentile | 0.909 (0.741-0.970) |
| Mean | 0.996 (0.989-0.999) |
| Median | 0.989 (0.966-0.996) |
| Kurtosis | 0.910 (0.752-0.970) |
| Skewness | 0.945 (0.841-0.982) |
| D* |  |
| 10th percentile | 0.993 (0.979-0998.) |
| 90th percentile | 0.946 (0.846-0.982) |
| Mean | 0.923 (0.785-0.974) |
| Median | 0.981 (0.943-0.994) |
| Kurtosis | 0.985 (0.953-0.995) |
| Skewness | 0.984 (0.954-0.995) |
| *f* |  |
| 10th percentile | 0.975 (0.927-0.992) |
| 90th percentile | 0.998 (0.992-0.999) |
| Mean | 0.995 (0.984-0.998) |
| Median | 0.993 (0.980-0.998) |
| Kurtosis | 0.963 (0.892-0.988) |
| Skewness | 0.976 (0.931-0.992) |
| Alpha |  |
| 10th percentile | 0.960 (0.885-0.987) |
| 90th percentile | 0.991 (0.972-0.997) |
| Mean | 0.920 (0.777-0.973) |
| Median | 0.934 (0.813-0.978) |
| Kurtosis | 0.970 (0.911-0.990) |
| Skewness | 0.948 (0.846-0.983) |
| DDC |  |
| 10th percentile | 0.989 (0.966-0.996) |
| 90th percentile | 0.999 (0.997-1.000) |
| Mean | 0.998 (0.995-0.999) |
| Median | 0.965 (0.898-0.989) |
| Kurtosis | 0.593 (0.107-0.849) |
| Skewness | 0.718 (0.315-0.901) |


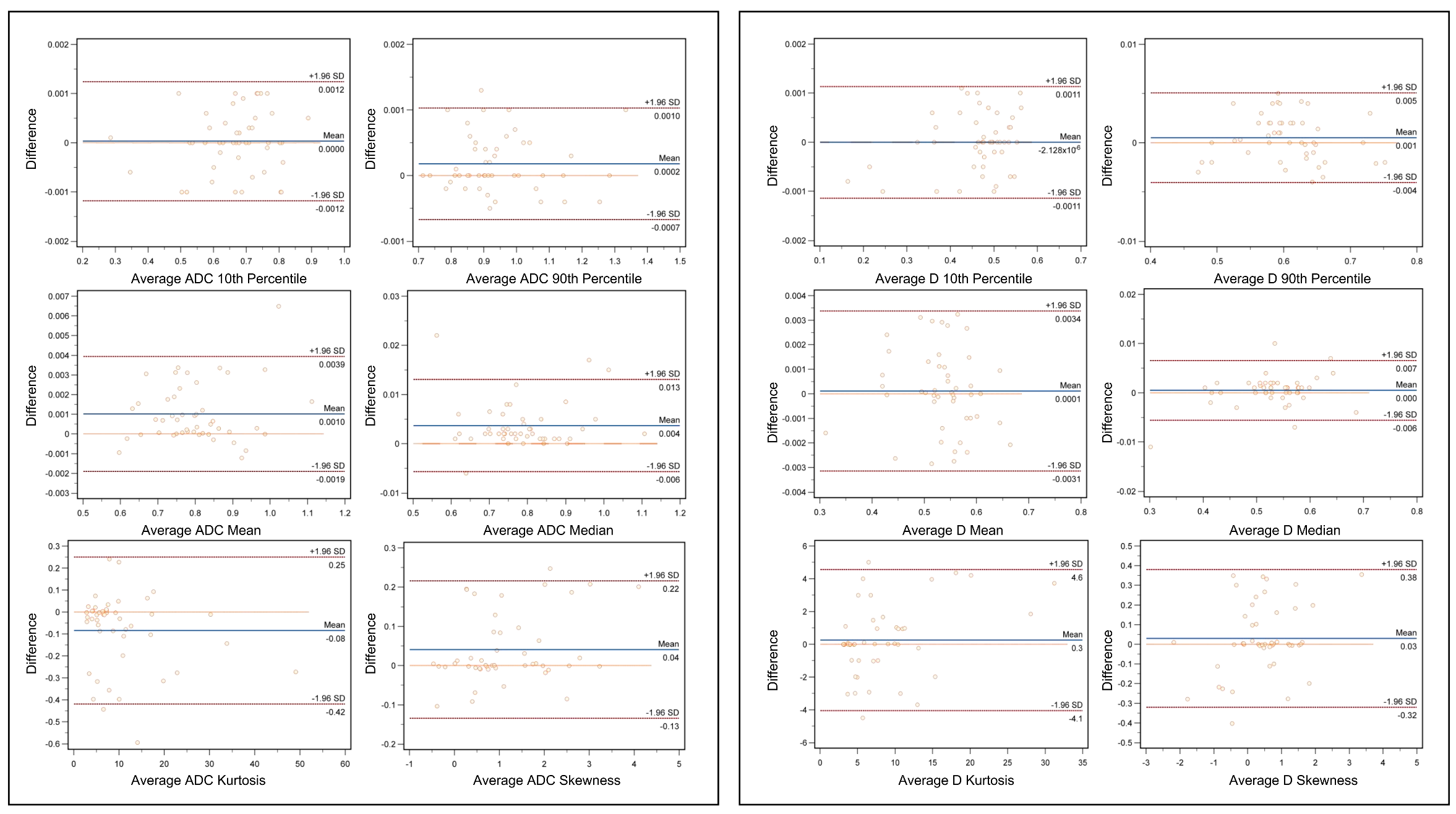


Figure S1.1 Bland‒Altman Plots of ADC and D histogram parameters.


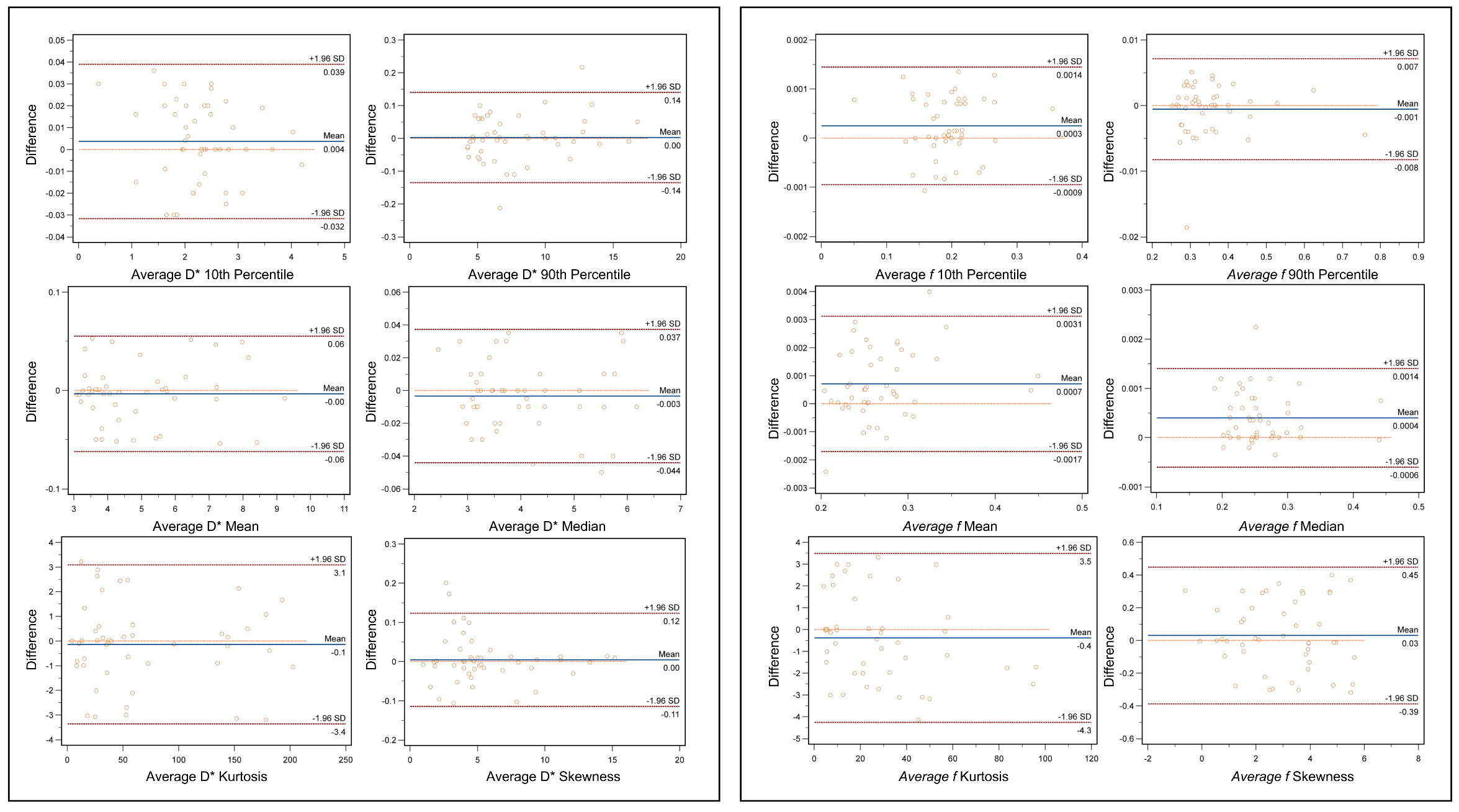


Figure S1.2 Bland‒Altman Plots of D* and *f* histogram parameters.


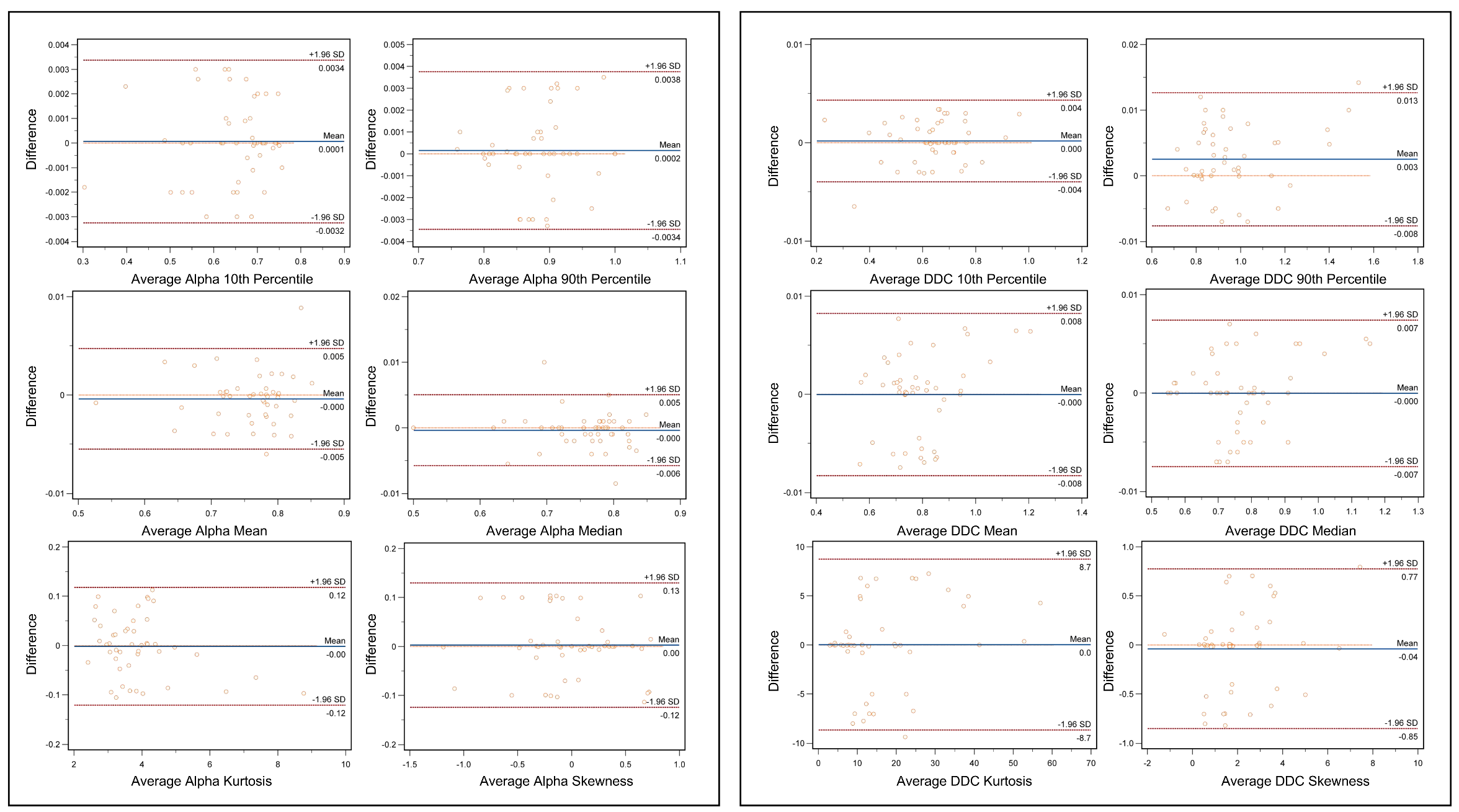


Figure S1.3 Bland‒Altman Plots of alpha and DDC histogram parameters.


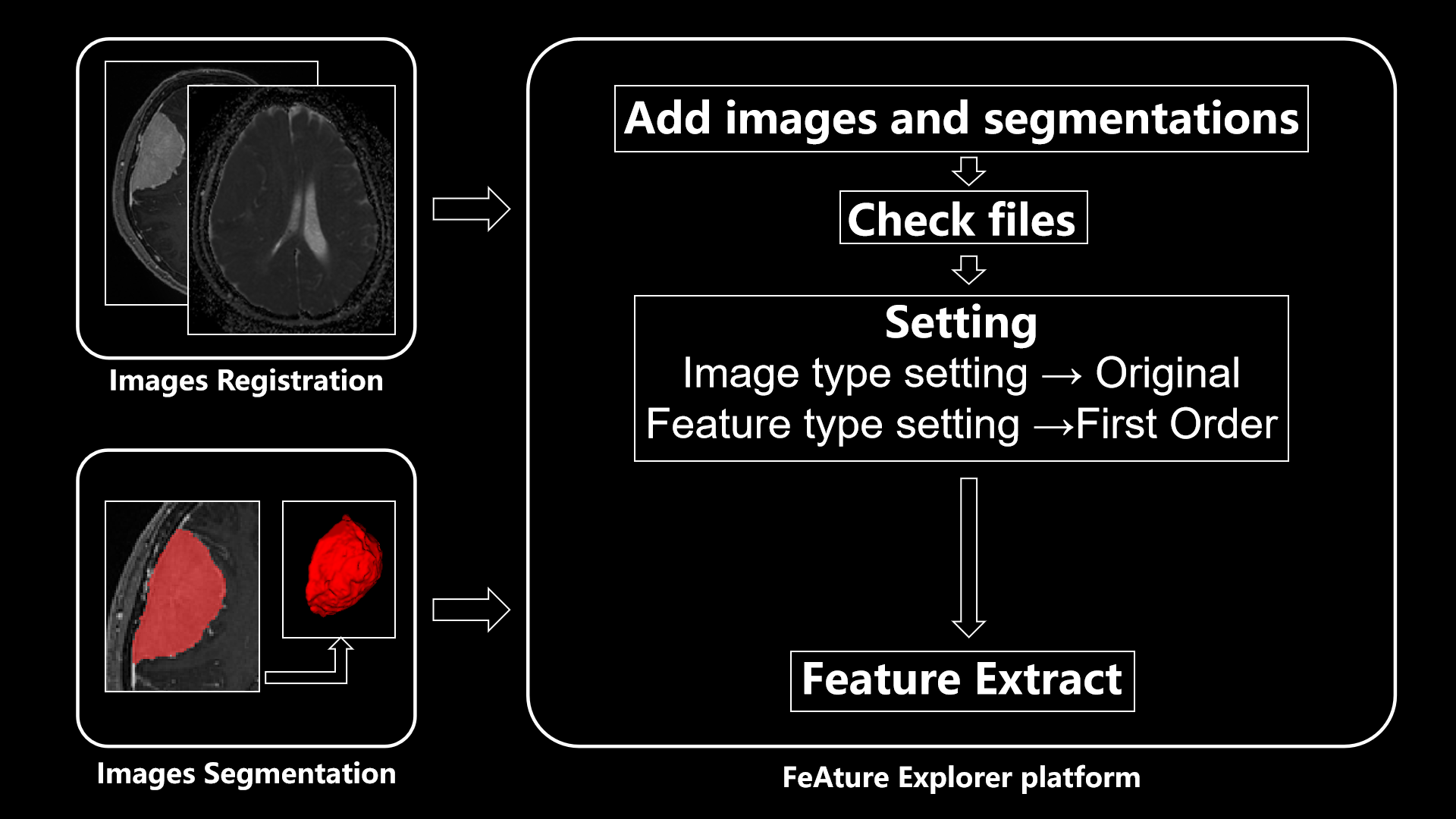


Figure S2 Workflow chart of histogram analysis.
